# Synergistic Processing of Biphenyl and Benzoate: Carbon Flow Through the Bacterial Community in Polychlorinated-Biphenyl-Contaminated Soil

**DOI:** 10.1038/srep22145

**Published:** 2016-02-26

**Authors:** Mary-Cathrine Leewis, Ondrej Uhlik, Mary Beth Leigh

**Affiliations:** 1 Institute of Arctic Biology, University of Alaska Fairbanks, Fairbanks, AK, USA; 2 Department of Biochemistry and Microbiology, Faculty of Food and Biochemical Technology, University of Chemistry and Technology, Prague, Czech Republic

## Abstract

Aerobic mineralization of PCBs, which are toxic and persistent organic pollutants, involves the upper (biphenyl, BP) and lower (benzoate, BZ) degradation pathways. The activity of different members of the soil microbial community in performing one or both pathways, and their synergistic interactions during PCB biodegradation, are not well understood. This study investigates BP and BZ biodegradation and subsequent carbon flow through the microbial community in PCB-contaminated soil. DNA stable isotope probing (SIP) was used to identify the bacterial guilds involved in utilizing ^13^C-biphenyl (unchlorinated analogue of PCBs) and/or ^13^C-benzoate (product/intermediate of BP degradation and analogue of chlorobenzoates). By performing SIP with two substrates in parallel, we reveal microbes performing the upper (BP) and/or lower (BZ) degradation pathways, and heterotrophic bacteria involved indirectly in processing carbon derived from these substrates (i.e. through crossfeeding). Substrate mineralization rates and shifts in relative abundance of labeled taxa suggest that BP and BZ biotransformations were performed by microorganisms with different growth strategies: BZ-associated bacteria were fast growing, potentially copiotrophic organisms, while microbes that transform BP were oligotrophic, slower growing, organisms. Our findings provide novel insight into the functional interactions of soil bacteria active in processing biphenyl and related aromatic compounds in soil, revealing how carbon flows through a bacterial community.

Polychlorinated biphenyls (PCBs) are a class of persistent organic pollutants that were widely used in such industrial applications as heat transfer fluids and electrical insulators until their production was banned globally in the 1980s. It is estimated that almost ten million kilograms still persist in the environment^1^. High-level PCB contamination has been reported at over 111 sites around the state of Alaska (http://dec.alaska.gov/) and at over 8770 sites across the U.S. (http://www.epa.gov/epawaste/hazard/tsd/pcbs/pdf/national.pdf). *Ex situ* physical-chemical remediation strategies or landfilling are commonly used for PCB-contaminated soils, while bioremediation using microorganisms and plants has potential to be applied either *ex situ* or *in situ*. Cleanup of sites through physical removal of soils and transport to a treatment facility can be extremely costly and result in much of the contaminant left behind in association with loose soil. Bioremediation is a potentially less expensive alternative for contaminated soil cleanup, yet remains challenging for several reasons such as differential recalcitrance of different congeners, extended times required to achieve regulatory cleanup limits, and concerns about the potential for degradative intermediates to remain^2^,^3^,^4^.

For more than three decades, the aerobic microbial degradation of PCBs, its analogue biphenyl (BP)^5^ and its degradation intermediate benzoate (BZ) has been the subject of a large body of research^6^. The aerobic biodegradation of (chloro)biphenyl to (chloro)benzoate and 2-hydroxypenta-2,4-dienoate, termed the upper PCB degradation pathway, is characterized by four separate reactions depicted in Fig. 1 6,7,8. (Chloro)BZ may be further metabolized via catechol and the β–ketoadipate pathway and funneled into central metabolism^6^,^9^. (Chloro)BP and (chloro)BZ can also be degraded via cometabolism, a process in which microbes metabolize chlorinated compounds fortuitously using enzymes synthesized to degrade the primary substrates, in this case BP or BZ^10^,^11^,^12^. Some microorganisms, such as certain *Arthrobacter, Pseudomonas, Rhodococcus* and *Burkholderia*, are capable of performing complete mineralization of BP along with some (chloro)BP^1^,^12^,^13^ and growing on a range of chlorinated substrates such as chlorinated BZ and BP^5^,^13^,^14^. Other organisms are known to possess only the upper pathway and/or to generate dead-end metabolites of BP^6^,^15^. BZ catabolic pathways such as the β-ketoadipate pathway are relatively widespread in the soil environment^9^. In the context of the contaminated soil environment, it is not known if BP is mineralized largely by the same group of organisms possessing both the upper and lower pathways, or if it is processed synergistically by separate microbial guilds specializing in BP and BZ biodegradation. Knowing this could reveal novel insights into community interactions during processing of PCBs and other aromatics (such as plant-associated compounds) and aid in efforts to stimulate relevant organisms during bioremediation^16^,^17^ or phytoremediation, since plant-associated compounds, such as phenols including flavonoids, have been shown to induce bioremediation of contaminated soils and microbial processing of PCBs^7^,^18^,^19^.

Microbial ecologists have long sought to understand the mechanisms underlying microbial community processing of complex carbon (C) substrates. Stable isotope probing (SIP) methods^20^,^21^,^22^ have greatly advanced understanding of which organisms within a complex community are active in degrading certain substrates, whether directly or indirectly. To date, SIP studies have provided valuable insight into the processing of C from individual substrates or mixtures of compounds^23^,^24^,^25^,^26^. SIP also has great potential to reveal how members of the microbial community interact to degrade complex substrates and to show how C from a complex substrate flows through different microbial guilds over time^27^,^28^.

Because bacteria mediate biodegradative processes in contaminated environments, it is valuable to identify the microorganisms involved and elucidate their interactions and ecological growth strategies. This study aims to reveal information concerning synergistic biodegradation and C flow in weathered PCB-contaminated soil from Kodiak, Alaska, USA. We employed DNA-SIP in parallel incubations with two different substrates to identify the microbes performing the upper (BP) and lower (BZ) PCB biodegradation pathways. We then used bacterial 16S rRNA gene sequencing to identify the individual bacterial taxa that derived C from the substrates over time, including those that actively assimilated C from both substrates. We hypothesized that since not all bacteria possess genes for the upper and lower pathways, (i) a portion of the bacterial community will be active only in either the upper or lower pathway of BP degradation, however (ii) a subset of the community will be able to complete the entire degradation pathway, and (iii) given the widespread nature of BZ degradation genes and potential for faster gain of C and energy for the organism, a larger proportion of the community will be involved in BZ degradation than BP degradation. By using non-halogenated compounds that are standard proxies for PCBs and chlorobenzoates^12^,^17^,^29^,^30^, our study is relevant not only to contaminant biodegradation but also to processing of naturally occurring aromatics that are important to soil carbon cycling.

## Results

Within 1 day of incubation, excess ^13^CO_2_ was detected in microcosm headspaces containing labeled substrates and increased in abundance throughout the experiment (14 days). No change in ^13^CO_2_ was detected in control microcosms containing unlabeled BP or BZ. The amount of ^13^C-labeled substrate mineralized by the microbial community was calculated based on the amount of ^13^CO_2_ evolved (Table 1)^31^. In the ^13^C-BP incubated soils, approximately 1.81 μg (0.18% of total BP added) of ^13^C was mineralized within one day, and up to 5.91 μg (0.59%) within 14 days. In ^13^C-BZ-incubated microcosms, the amount of ^13^C mineralized was approximately 5.43 μg (0.54%) within one day and reached 127.8 μg (12.78%; 20-fold higher levels than for ^13^BP) within a 14-day incubation.

Q-PCR analyses of gradient fractions were conducted to examine the relative abundance of 16S rRNA gene throughout the gradient and to identify fractions containing ^13^C-labeled DNA (Supplementary Fig. S1). There was a substantial peak of DNA at buoyant density (BD) of 1.576 g.ml^−1^, which corresponds to the BD of unlabeled (predominantly ^12^C) DNA. In ^13^C-incubated samples, a second peak at BD greater than 1.599 g/ml was detected indicating the presence of ^13^C -DNA. Based on the q-PCR results, we combined the fractions with BD of 1.584–1.628 g.ml^−1^ to create a compiled ‘heavy’ fraction for each sample prior to downstream molecular analyses as density gradients run in duplicate for each sample were similar in DNA quantity and distribution (Supplementary Fig. S1). Equivalent fractions were compiled from unlabeled controls and were analyzed identically to detect any unlabeled bacterial DNA contamination in heavy fractions. Mann-Whitney U tests on T-RFLP data indicated that the replicate samples were not significantly different, allowing for the combination of replicates for down-stream pyrosequencing analysis. Only a small subset of peaks in the T-RFLP profiles were found in the heavy fractions from control DNA, and fragments of the same size were not major contributors to T-RFLPs in the heavy fractions from ^13^C-incubated samples. To identify the bacterial populations in the total community and active in assimilating C from labeled substrates, we sequenced 16S rRNA gene amplicons. The biphenyl early (day 04) sample did not achieve sufficient labeling to distinguish the community from unlabeled controls according to ANOVA and post-hoc Tukey comparison of means (Supplementary Table S1) and was excluded from further analyses. The numbers of pyrosequencing reads of labeled DNA in the samples selected ranged from 5684 to 14848 per sample after sequence processing (Table 2). Despite an intense sequencing effort, the total diversity was not fully sampled as was indicated by rarefaction curves (Supplementary Fig. S2).

**Figure 1:**
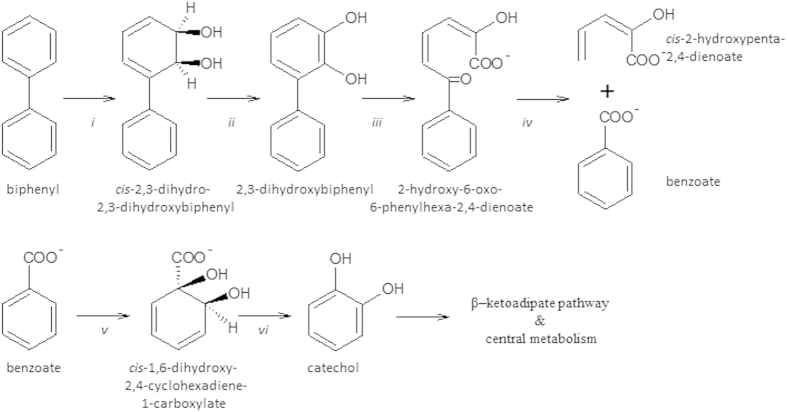
The upper (chloro)biphenyl degradation pathway and lower transformation of (chloro)benzoic acid to catechol. The enzymes involved in each reaction step are biphenyl 2,3 dioxygenase (i), 2,3-dihydro-2,3-dihydroxybiphenyl dehydrogenase (ii), 2,3-dihydroxybiphenyl 1,2-dioxygenase (iii), 2-hydroxy-6-oxo-6-phenylhexa-2,4-dienoate hydrolase (iv), benzoate 1,2-dioxygenase (v), and *cis*-diol dehydrogenase (vi).

The abundance of biphenyl dioxygenase genes (*bphA*), the gene that initiates the upper pathway of biphenyl degradation (Fig. 1), was quantified in total soil DNA and in heavy (labeled) fractions by q-PCR. The relative abundance of BphA gene, normalized by the total ^13^C-DNA concentration in each sample, was lowest in the total, unlabeled, soil community DNA (1.66 × 10^−3^ gene copies per ng DNA^−1^). Bacterial *bphA* abundance in heavy fractions obtained following ^13^C-BZ SIP was initially ten-fold higher than in the total soil DNA, and decreased in quantity over the time course (d01 BZ: 1.48 × 10^−2^ gene copies per ng ^13^C-DNA^−1^; d04 BZ: 8.15 × 10^−3 ^gene copies per ng ^13^C-DNA^−1^). In ^13^C-BP labeled samples, *bphA* abundance was 5.64 × 10^−3 ^gene copies per ng ^13^C-DNA^−1^ after 14 days, which was approximately five-fold higher than in total soil DNA and corresponded to d04 BZ *bphA* quantity.

Phylogenetic affiliations of pyrosequencing reads from ^13^C-DNA revealed diverse bacterial populations that were involved either directly or indirectly in acquiring C derived from BP or BZ (Supplementary Table S2). *Actinobacteria* predominated in the total soil bacterial community and in libraries obtained from incubations containing ^13^C-labeled BZ on day 4, and BP on day 14. *Proteobacteria* were present in all of the examined time points, but predominated in the BZ day 1 sample, where they accounted for 36.5% of total pyrosequencing reads. In addition, the phyla *Acidobacteria, Actinobacteria, Gemmatimonadetes,* and *Nitrospira* were found in all labeled DNA samples. In the early (day 1) ^13^C-BZ incubations, sequences of the order *Rhizobiales* predominated the labeled DNA, whereas *Burkholderia* and *Actinomycetales* dominated after 4 days of ^13^C-BZ. After 14 days, labeled sequences from ^13^C-BP samples were dominated by *Actinobacteria*. Some pyrosequencing reads could not be assigned by the RDP classifier, with 16.6% of the total community, 5.1-6.0% of the ^13^C-BZ, and 4.79% of ^13^C-BP remaining unclassified.

Bacteria labeled during incubation with ^13^C-BZ increased in richness over time (#OTUs and Chao1), but decreased in diversity index values over time, and bacterial sequences associated with the four-day incubation had the lowest diversity of all measured populations (Table 2). Species evenness shifted across the labeled populations over time, causing the reduction in diversity indices, which take into consideration both richness and evenness, but caused no reduction in richness (i.e. number of OTUs). Comparatively, the diversity of bacteria that derived C from ^13^C-BP, as measured by Chao1, Inverse Simpson and effective number of species, was higher than that measured in either of the ^13^C-BZ- incubated samples.

**Figure 2:**
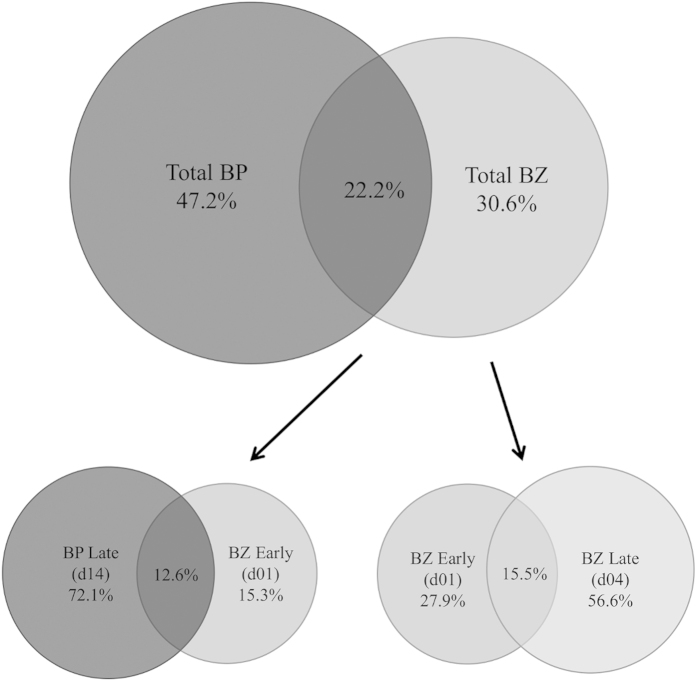
Overlap of OTUs across substrates. Venn diagrams showing the overall overlap of OTUs that derived C from the two labeled substrates at different time points, and the overlap in total labeled OTUs from each substrate. The percentages represent the percent of OTUs unique to each of the individual time points and/or substrate. Sequences were grouped at 3% dissimilarity.

To investigate the overlap in bacterial taxa that utilized the two substrates, the percentage of unique and shared OTUs (defined at 97% sequence similarity) that derived C from the different substrates and time points was analyzed and is presented using Venn diagrams (Fig. 2). When all OTUs present in ^13^C-DNA from BP and BZ were considered, 22.2% of the OTUs were shared in labeled DNA from both substrates. In ^13^C-BZ incubated soils, 15.5% of sequences were shared among early (day 1) and late (day 4) time points, and the early harvested soils (day 1) had fewer (27.9%) unique OTUs than the late harvested (day 4) soils with 56.6% unique OTUs. To examine the potential transition between the upper and lower portions of the BP degradation pathway, we compared percentages of OTUs present in both the late (day 14) ^13^C-BP and early ^13^C-BZ (day 1) incubated soils: 12.6% of OTUs were shared between the two groups.

**Figure 3:**
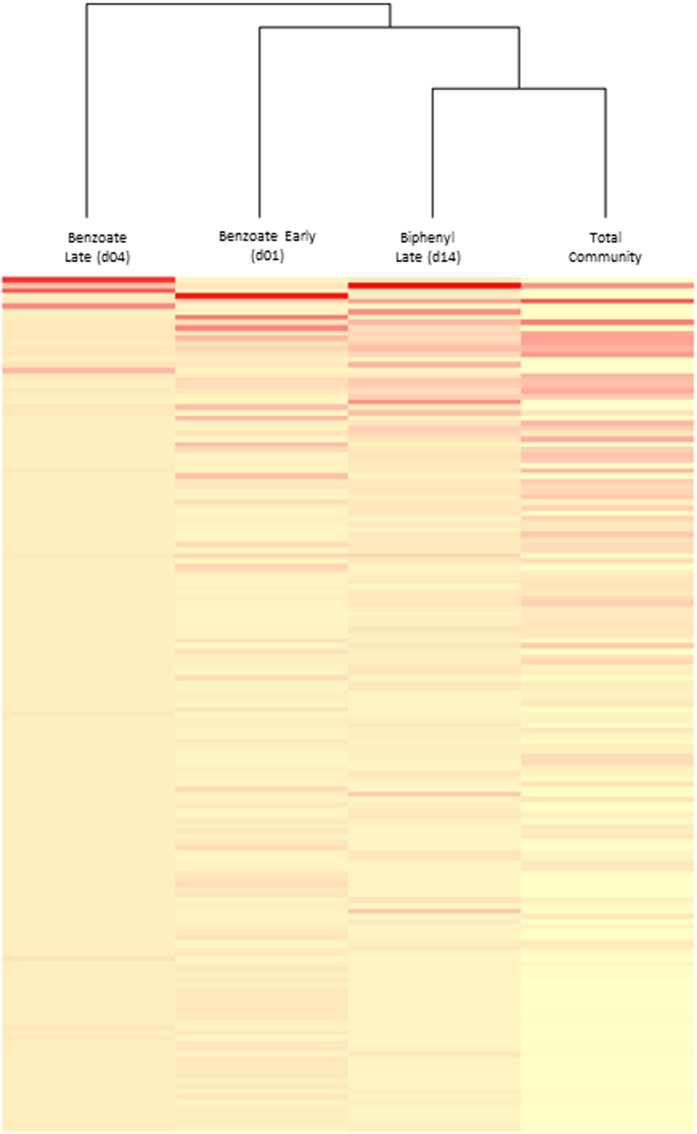
Clustering and heat map of OTUs. Heat map and dendrogram generated using the relative abundance and overlap of OTUs which represent more than 0.2% of the total population in each sample. The color code indicates relative abundance, ranging from light yellow (low abundance) to dark red (high abundance). The dendrogram was generated using average linkage hierarchical clustering based on a Bray-Curtis distance measure.

The relative abundance and overlap of OTUs that represent more than 0.2% of the total population in each sample were analyzed and visualized using a heat map and dendrogram (Fig. 3). The community that was labeled at the later ^13^C-BZ time point was the most unique, while that from the early incubated (day 1) ^13^C-BZ was more similar to both the late ^13^C-BP and total communities.

To investigate the phylogenetic differences and overlaps between the most dominant utilizers of BZ and BP, we generated a table for the top ten most abundant OTUs from each incubation and time point (Table 3). In soils incubated with ^13^C-BP after 14 days of incubation the dominant taxa were most similar to *Arthrobacter oryzae* (OTU 2, 12%), *Thermosipho geolei* (OTU 7, 5%), *Pseudomonas lutea* (OTU 24, 4%), *Acidothermus cellulolyticus* (OTU 5, 3%), and *Rhodanobacter soli* (OTU 17, 2%). In soils incubated with ^13^C-BZ, a smaller set of OTUs became highly abundant over the course of the incubation than was observed in the ^13^C-BP sample. By day one (early), dominant labeled OTUs in the ^13^C-BZ incubation were *Methylobacterium jeotgali* (OTU 4), which comprised 19% of the total labeled sequences, and *Rhodococcus qingshengii* (OTU 8), which represented 11% of sequences. The late (four days) incubated ^13^C-BZ soils were highly dominated by sequences most similar to *Burkholderia phytofirmans* (OTU 1, 34%), *Burkholderia sediminicola* (OTU 3, 27%), and *Cupriavidus basilensis* (OTU 6, 16%).

## Discussion

With this study, we sought to better understand how C from aromatic compounds flows through the soil microbial ecosystem. Using parallel SIP incubation studies, the experiment revealed taxa that participate in the assimilation of C from the polyaromatic compound BP (an unchlorinated analogue of PCBs) and BZ (product of partial BP degradation, analogue for chlorobenzoates and common monoaromatic in the environment) in a PCB-contaminated soil, and how they respond and work in synergy to process these substrates. An array of bacterial taxa derived C from biodegradation of ^13^C-BP and/or ^13^C-BZ, either directly or indirectly through cross feeding. Copy numbers of the BphA gene were enriched in labeled DNA from both BZ and BP incubations beyond that of total soil community DNA, supporting the conclusion that labeled DNA represented organisms truly active in aromatic biodegradation rather than exclusively contaminants or cross-feeders. The majority of taxa that assimilated the ^13^C-labeled aromatics appeared to perform either BP or BZ utilization, but not both. A subset of organisms derived C from both substrates, which could be explained by three possible scenarios: i) they possess both BP and BZ pathways, and thus can mineralize both substrates, ii) they are BZ utilizers that scavenge BZ released by BP-utilizers or iii) they scavenge other substrates or dead biomass generated by BP- and BZ-utilizing microbes. Through this time-course study, we observed a difference in ecological growth strategy for the two microbial guilds, with fast growing bacteria performing BZ degradation, and slower growing microbes degrading BP.

The rapid response to labeled BZ by the microbial community indicates that microbes involved in the lower pathway of BP degradation (BZ degradation) may be adapted to quickly react and utilize ^13^C-BZ when it is present in the environment, indicative of a copiotrophic population. Of the bacteria that assimilated BZ-carbon, all but one (early OTU 8; late OTU 2) of the top five most abundant OTUs from each time point have been described as copiotrophic microbes^32^,^33^. The initially lower rates of ^13^CO_2_ evolution in ^13^C-BP-incubated microcosms might indicate that the BP-utilizing populations could be classified as “oligotrophic” and adapted for the optimal utilization of recalcitrant C, such as BP. Of the five most abundant OTUs from both BP early and late incubations, half have been previously described as oligotrophic populations (Table 3; Fierer *et al.* 2007). Also, approximately 49% of the BP incubated populations were Gram-positive, or mostly associated with oligotrophic microorganisms (Table 3, Supplementary Table S2).

Members of the genera *Pseudomonas* and *Arthrobacter,* implicated in BP metabolism in this study, have often been reported to be associated with the upper pathway of BP degradation, and less frequently the lower pathway, and are known to contain the *bph* operon and degrade PCBs^1^,^6^,^12^,^17^,^23^,^34^. Pseudomonads have previously been reported to be the most prevalent group of bacteria in the biodegradation of complex organic compounds^35^. Both the *Acidimicrobiales* and *Marmoricola* have not been previously identified in the metabolism of BP or PCBs. The closest RDP match for the unclassified *Acidimicrobiales* was *Terriglobus saanensis* (Table 3), a bacterium isolated from tundra soils^36^. Although the ecological roles of many acidobacteria are poorly understood^37^, the *Terriglobus* species has wide potential physiological capabilities in soils including hydrolysis of different polysaccharides such as starch, pectin, laminarin and aesculin. Among organisms that derived C from BZ, the genera *Rhodococcus, Burkholderia,* and *Cupriavidus* have all previously been implicated in PCB degradation. Additionally, the genus *Propionibacterium,* which derived C from BZ, has previously been reported to use various aromatic hydrocarbons such as benzene, biphenyl, toluene, and styrene as sole carbon and energy sources^38^.

We investigated the diversity and overlap of organisms that derived C from each substrate over time to elucidate the flow of C through the community and to determine how the guilds may interact (Figs 2 and 3). While a relatively small number of labeled OTUs increased dramatically in relative abundance in ^13^C-BZ labeled populations during the incubation, increasing numbers of unique, but low-abundance, OTUs over time were also detected, suggesting that some members of the community were slower-growing organisms, or that C flowed out into the community via secondary feeding.

Most taxa that became labeled were involved in either BP or BZ degradation but not both, based on 16S rRNA analyses. Uhlik *et al.* (2012) found that, in legacy contaminated soil from the former Czechoslovakia, the majority of taxa involved in BP and BZ degradation overlapped. When all of the OTUs detected in this study in ^13^C-BP and ^13^C-BZ incubated soils were compared, 16% of OTUs were shared between the two substrates (Fig. 2), suggesting that the majority of the biodegradative microbial populations do not utilize both the upper and lower BP degradation pathways; rather the pathways occur primarily in separate populations. However, *BphA* genes were enriched in labeled DNA from both BP and BZ, further supporting the observation that BP-degradation capabilities do exist within a portion of the BZ-utilizing guild. Thirteen percent of the OTUs were shared between late ^13^C-BP and early ^13^C-BZ, with more unique OTUs present in late ^13^C-BP soils (72.1%; Fig. 2). At most, 12.6% of BP-degrading taxa may possess both pathways, but the number is likely lower given that cross-feeding on metabolites or dead biomass of BP-degraders may account for some portion of the overlap (Fig. 2; DeRito *et al.* 2005). The overlapping OTUs were mostly associated with the phylum *Proteobacteria*, which has been implicated in both BP and BZ degradation, but also contains known heterotrophs and potential cross feeders^23^,^26^. Taxa similar to the OTU in the ^13^C-BP labeled community, *Methylobacterium jeotgali* (OTU 4), have previously been found as sequencing contamination, however because this OTU represents 19% of the labeled community in BP-incubated samples, and was not found in the other ^13^C-incubated samples, it does not appear to be a contaminant in this study^39^. Additionally, methane- and ammonia-oxidizing microbial communities, such as *Methylobacterium jeotgali*, were also previously hypothesized to co-metabolize PCBs in soils^40^.

The bacterial guilds that assimilated C from each of the substrates showed very different rates of substrate utilization and population shifts over the course of the incubations. Soil consortia that assimilated each of the two substrates differed in their patterns of substrate mineralization (^13^CO_2_ evolution); in which those mineralizing BP did so at a much slower rate than those mineralizing BZ, based on of ^13^CO_2_ evolution (Table 1). The BZ utilizing bacterial community became highly dominated by relatively few (1–2) OTUs throughout the course of the incubations, and BP-assimilating bacteria were relatively much more diverse and OTU-rich (Table 2). Although the observed differences in ^13^C processing among the guilds could be due to differences in bioavailability of BP versus BZ, the observed differences also suggest that communities possess diverse ecological patterns of growth.

Recent work recognizing broad, ecologically-based, classes of bacteria^32^,^41^,^42^ could explain the differences in substrate processing rates and the diversity of organisms that dominate the assimilation of the more recalcitrant (BP) versus the more labile (BZ) substrates. These ecologically defined classes are similar to the r-K scheme which, in general, states that r-strategists are adapted to high rates of reproduction and K-strategists are adapted for optimal utilization of environmental resources^43^. When applied to microbial ecology, r-strategists are defined as ‘copiotrophic’ bacteria or organisms which show an increase in numbers in response to the addition of labile C sources to soil and K-strategists are defined as ‘oligotrophic’ bacteria which do not show a marked increase in numbers in response to the addition of a labile C source to the soil. It has been found through experimentation, observation, and meta-analytical approaches that Gram-negative bacteria, such as members of the phylum *Bacteroidetes* and subphyla Beta- and Alpha-proteobacteria, which rapidly colonize and grow under labile C-rich conditions, are copiotrophs. Gram-positive soil bacteria, such as some *Actinobacteria*, as well as *Acidobacteria* tend to be more successful in resource-limited situations and are considered oligotrophs^32^,^44^.

The substrate utilization data, as measured by ^13^CO_2_ evolution (Table 1), provide one insight to the ecological growth strategies of the microorganisms associated with each of the substrates. The microcosms incubated with BZ showed a faster rate of ^13^C–labeled substrate mineralization than the BP-incubated microcosms (Table 1). This difference could be explained by the structural differences between BP and BZ, with BP having a more complex structure. The fact that the BP treatments contained approximately twice the molar quantity of ^13^C atoms underscores the notably higher rate of BZ substrate mineralization rate compared to BP. It has been found that compounds that require a large number of enzymatic steps to be degraded and are biochemically expensive to degrade, such as BP, may result in lowered substrate use efficiency^45^. For example, in some soil systems, a 50% loss of PCBs was reported after a five month incubation, whereas a similar loss was reported within 50 days for some chlorobenzoate enrichment cultures^46^,^47^.

Another indication of differential growth strategies is evidenced in the shifts in populations associated with the two incubated substrates (Table 2). During the rapid utilization of BZ, a smaller number of BZ utilizers appeared to rapidly grow and dominate substrate assimilation over time than was observed during BP utilization. This is evident in the effective number of species in ^13^C-BZ incubated microcosms, which decreased over time (d01 to d04). Although Chao1 increased over time, diversity decreased when evenness was taken into account (effective number of species, Simpson Index), and the species that were detected increased in relative abundance. In contrast, the bacteria that assimilated BP-derived carbon were considerably more OTU-rich and mineralized the substrate at a much lower rate than for BZ degraders. These observations suggest that BP-metabolizing microorganisms are slower-growing populations than BZ utilizers, and slowly process recalcitrant substrates, both of which are characteristics of oligotrophic organisms.

Using a parallel substrate SIP study design, we revealed that the biotransformation of the aromatics BP and BZ in a PCB-contaminated system was mainly performed by distinct bacterial guilds with different ecological growth strategies. Our results suggest that the microbial populations associated with BZ are mostly fast-growing, copiotrophic organisms, while microbes that transform BP are oligotrophic, slower-growing, organisms. The different patterns of use of the available C sources could represent adaptations for the efficient use of monoaromatic versus polyaromatic substrates. A small but substantial proportion of the community assimilated C from both substrates, which may indicate that they possess both the upper and lower BP pathways, or that they are effective cross-feeders and scavengers of labeled metabolic products. Future studies should focus on the ^13^C-labeled metabolites produced by BP- and BZ-utilizing populations over time to examine the transfer of metabolites between microbial guilds during degradation of biphenyl-containing compounds. Because BP and BZ are analogues for PCBs and chlorobenzoates, respectively, these findings have implications for PCB biodegradation. The findings imply that guilds responsible for initial biodegradation of PCBs to chlorobenzoates are more of a limiting factor than chlorobenzoate degraders in terms of responsiveness to contaminants and biodegradation activity, and that biostimulation efforts targeting initial PCB-degraders may have the most benefit. Further studies should investigate synergistic biodegradation of these compounds with the added challenges of chloro substitutions.

## Materials and Methods

### SIP Microcosms

PCB-contaminated soils for this experiment were collected in clean sterile glass jars from the Coast Guard Station at Drury Gulch, Kodiak Island, Alaska, USA as a portion of cleanup actions in accordance with Federal Comprehensive Environmental Response, Compensation, and Liability Act of 1980 (CERCLA, http://dec.alaska.gov/spar/csp/ dod_sites.htm), and stored at 4 °C until analysis. The calculated total PCB concentration in investigated soils was 32.53 mg kg^−1^ as determined using Soxhlet extraction and run on an Agilent (Santa Clara, CA, USA) 6890N GC equipped with an ECD detector according to the method reported by Slater *et al.* (2011).

Microcosms for SIP incubations were established in sterile 125 mL glass serum bottles (Wheaton, Millville, NJ, USA). Uniformly labeled ^13^C-BP (^13^C_12_H_10_), ^13^C-BZ (^13^C_7_H_5_O_2_; both 99% labeled; Isotec, Miamisburg, OH, USA), unlabeled BP, or unlabeled BZ were dissolved in acetone and an appropriate volume was added to each microcosm by pipetting the solution onto the inside of the serum bottle and allowing the acetone to evaporate, leaving behind 1 mg BP or BZ crystals on the walls of the serum bottles^23^. After the BP or BZ solutions had dried fully, 7 g of homogenized contaminated soil and 400 μL sterile water was added to each serum bottle. Bottles were then sealed with a Teflon stopper and aluminum crimp top. Duplicate microcosms were set up for each substrate and each incubation time point (1, 4 and 14 days). These time points were selected based on times previously shown to reveal BP-degrading bacteria using SIP^23^,^26^. Microcosms were incubated at room temperature (22 °C) until destructive harvesting and soils were stored at −80 °C until further analysis^48^,^49^. An aliquot of soil without the addition of ^13^C labeled substrate was frozen at −20 °C to serve as the time 0 (T0) sample.

### Headspace Gas Isotope Analysis

One milliliter of headspace gas was collected at microcosm initiation and at each sampling time point using an N_2_-purged syringe to quantify mineralization of labeled substrates. CO_2_ was analyzed for δ^13^C within seven days on a Trace Gas system interfaced to an IsoPrime mass spectrometer (GV instruments, Manchester, UK) at the Alaska Stable Isotope Facility (University of Alaska Fairbanks) as previously described^23^,^24^. The mineralization of ^13^C-labeled BP or BZ to ^13^CO_2_ was calculated based on the assumption that in the aerobic incubation, oxygen was the terminal electron acceptor for microbial degradation of the supplied substrate.

### ^13^C-DNA Isolation

Total soil DNA was extracted using the PowerMax soil DNA isolation kit (MoBio Laboratories, Inc., USA) using the standard protocol. After the final elution, DNA was concentrated according to Uhlik *et al.* (2012)^26^ to increase yield. All solutions were diluted to obtain a concentration of 100 ng/μl. Isopycnic centrifugation and gradient fractionation was performed according to the method of Uhlik *et al.* (2012)^26^. Fractions were frozen at −20 °C until further analysis.

To locate and quantify bacterial DNA in SIP fractions, q-PCR targeting bacterial 16S rRNA was performed on every fraction for each time point in duplicate as described previously^24^,^50^. Fractions that were determined to contain ^13^C-labeled bacterial DNA based on quantities of DNA in high density fractions that exceeded that of unlabeled controls were combined to create a combined “heavy DNA” sample for each gradient (buoyant density (BD) 1.584–1.628 g.ml^−1^). Equivalent heavy DNA samples were prepared for controls incubated with unlabeled substrates and they were subjected to the same downstream analyses as ^13^C-labeled DNA to identify and eliminate any background contamination present in controls.

### T-RFLP Analysis

Bacterial community profiling using T-RFLP was performed on heavy fractions (^13^C-DNA), control heavy fractions (^12^C incubated soils), and total community DNA to provide an initial screening of the diversity of labeled DNA, to determine if replicates were statistically different prior to down-stream sequencing, and to assess any background contamination in heavy fractions from unlabeled DNA prior to pyrosequencing. PCR reactions to amplify the 16S rRNA gene were carried out as described previously^51^,^52^. Terminal restriction fragments less than 50 bp and above 1000 bp in size were eliminated from all data sets.

### Phylogenetic identification

Amplification and pyrosequencing were performed by a modification of a method described previously by Engelbrektson *et al.* (2010)^26^. A portion of the 16S rRNA gene (positions 8 to 357 [V1 and V2], *Escherichia coli* numbering) was amplified using the 8F primer containing a Roche 454 A pyrosequencing adaptor, a 10 base pair unique bar code and 357R primer also containing a Roche 454 B pyrosequencing adaptor.

The PCR mixture (final volume, 25 μl) contained 1 μl each primer (10 μM), 0.5 μl dNTP mix (10 mM), 2.5 μl FastStart 10 X Buffer #2, 0.25 μl FastStart HiFi Polymerase (5 U/μl), and 18.75 μl molecular biology grade water (Roche). The following thermal cycling scheme was used: initial denaturation at 95 °C for 3 min and 30 cycles of denaturation at 95 °C for 30 sec, annealing for 1 min at 55 °C, and extension at 72 °C for 1.5 min, followed by a final extension period at 72 °C for 10 min. Each PCR product was obtained in three parallel reactions, the resulting preparations were mixed, purified using Pure-Link PCR purification kit (Invitrogen, USA) and pooled for downstream sequencing. Roche 454 GS FLX Titanium sequencing (454 Life Sciences, USA) was performed on pooled reactions at the Institute of Molecular Genetics, Czech Academy of Sciences, Prague, Czech Republic.

### Processing of Pyrosequencing Data

Raw pyrosequencing data (*.sff files) were processed using the mothur software package version 1.30.2 and following the standardized operating procedure^53^,^54^ with minor modifications as described previously^26^. Sequence control and analyses were performed on 16S rRNA genes from ^13^C-DNA from the BP microcosms day 4 and 14, BZ day 1 and 4 according to He *et al.*^26^,^26^. After sequencing, significant differences between labeled and unlabeled microbial communities present in heavy fractions were detected using one-way analysis of variance (ANOVA). Comparison of sample means was done using the Tukey post-hoc test at a significance level of P-value < 0.05. If the community from ^13^C-incubated samples were not significantly different from the Total Community or time 0 heavy fractions, these samples was excluded from further analyses. Pyrosequencing reads were deposited in the metagenomics (MG) - RAST server^55^ under the MG-RAST Project ID 8748.

### Q-PCR of *bphA*

The abundance of portions of genes coding for the α subunit of biphenyl dioxygenase (BphA) were amplified from ^13^C-DNA after SIP with an ABI 7900 HT Fast Real-Time PCR System (Applied Biosciences, USA). Reactions were performed in a 10 μl final reaction volume containing 5 μl of Kapa SYBR FAST qPCR Master Mix (Kapa Biosystems, USA), 1 μM of each primer, approximately 5 ng of ^13^C-DNA. The *bphA* sequences were amplified using a primers 512f (tgr tbt tyg cva ayt ggg a) and 674r (tcb gcn gcr aay ttc cag tt). The reverse primer is a reverse complement sequence of a previously used primer BPHD-f3^56^ and the forward primer was designed based on known sequences available in RDP FunGene database similarly as previously described^57^. The performance of the primers was verified using the genomic DNA of previously described PCB-degrading strains and the cycling conditions used were as follows: 3 min at 95 °C, 30 cycles of 5 s at 95 °C, 30 s at 56 °C, and 20 s at 72 °C, followed by a final extension at 72 °C for 5 min, and a melting curve.

## Additional Information

**How to cite this article**: Leewis, M.-C. *et al.* Synergistic Processing of Biphenyl and Benzoate: Carbon Flow Through the Bacterial Community in Polychlorinated-Biphenyl-Contaminated Soil. *Sci. Rep.* **6**, 22145; doi: 10.1038/srep22145 (2016).

## Supplementary Material

Supplementary Information

## Figures and Tables

**Table 1:** Mineralization of ^13^C-BP and ^13^C -BZ as determined by ^13^CO_2_ evolution.

SIP incubation time (days)	^13^CO_2_ excess in headspace (nmol)		^13^C substrate mineralized (μg)		^13^C substrate mineralized (%)	
	Benzoate	Biphenyl	Benzoate	Biphenyl	Benzoate	Biphenyl
1	311.3	141.1	5.431	1.814	0.543	0.181
4	4235	203.8	73.88	2.618	7.388	0.262
14	7327	458.8	127.8	5.895	12.78	0.590

**Table 2:** Diversity estimates and number of sequences obtained after raw sequence data processing and normalization.

Sample ID		Sequences after processing	# OTUs (0.03)	Chao	1/D (Inverse Simpson)	Effective Number of Species
Biphenyl	Day 14	5684	938	1348	44.5	268.6
Benzoate	Day 01	13474	310	553	16.6	54.1
	Day 04	14848	515	856	4.6	10.9
Total Community		9745	2174	4876	159.5	717.3

**Table 3:**
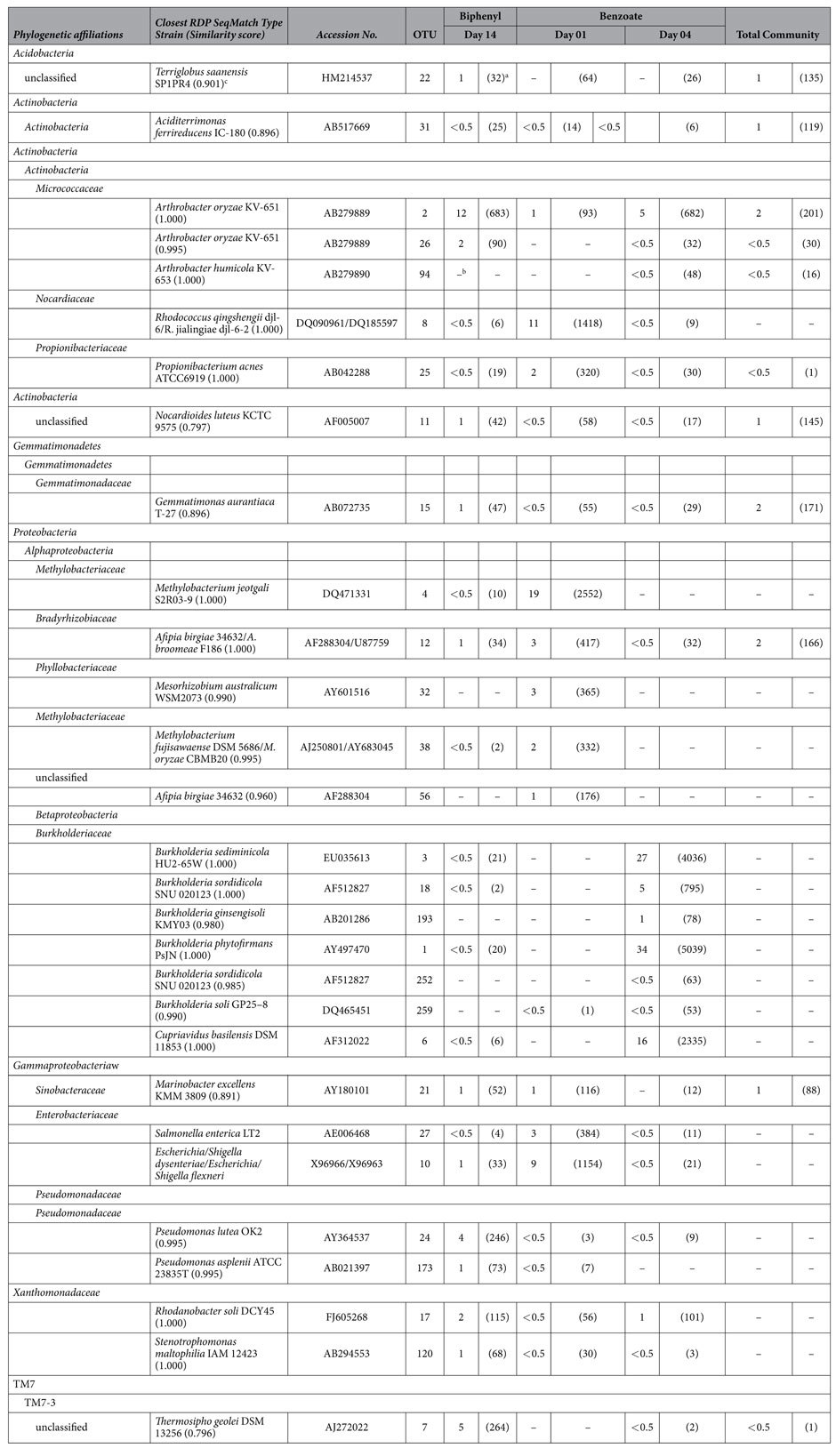
Top OTUs detected in ^13^C-DNA after incubation of soil with ^13^C-BP and ^13^C-BZ.

The percentage and number (in parenthesis) of sequences associated with each OTU is indicated under each investigated time point and substrate. Taxonomic assignments were generated using the Ribosomal Database Project’s classifier.

^a^The numbers of pyrosequencing reads assigned using the RDP classifier (80% confidence threshold) are shown in parentheses.

^b^Sequences represented <0.5% of total sequences present in the group.

^c^Nearest RDP match; similarity score shown in parentheses.

## References

[b1] Singer A. C., Gilbert E. S., Luepromchai E. & Crowley D. E. Bioremediation of polychlorinated biphenyl-contaminated soil using carvone and surfactant-grown bacteria. Appl. Microbiol. Biotechnol. 54, 838–843 (2000). 11152078 10.1007/s002530000472

[b2] Macková M. *et al.* In Geomicrobiology: Molecular and Environmental Perspective (eds. Loy A., Mandl M. & Barton L. L.) 347–366 (Springer, 2010).

[b3] Leewis M.-C., Reynolds C. M. & Leigh M. B. Long-term Effects of Nutrient Addition and Phytoremediation on Diesel and Crude Oil Contaminated Soils in subarctic Alaska. Cold Reg. Sci. Technol. doi: 10.1016/j.coldregions.2013.08.011 (2013). PMC3909700 24501438

[b4] Aken B. Van, Correa P. A. & Schnoor J. L. Phytoremediation of Polychlorinated Biphenyls: New Trends and Promises. Environ. Sci. Technol. 44, 2767–2776 (2010). 20384372 10.1021/es902514d PMC3025541

[b5] Koubek J., Mackova M., Macek T. & Uhlik O. Diversity of chlorobiphenyl-metabolizing bacteria and their biphenyl dioxygenases in contaminated sediment. Chemosphere 93, 1548–55 (2013). 24007621 10.1016/j.chemosphere.2013.07.073

[b6] Pieper D. H. & Seeger M. Bacterial metabolism of polychlorinated biphenyls. J. Mol. Microbiol. Biotechnol. 15, 121–38 (2008). 18685266 10.1159/000121325

[b7] Francova K., Macková M., Macek T. & Sylvestre M. Ability of bacterial biphenyl dioxygenases from Burkholderia sp. LB400 and Comamonas testosteroni B-356 to catalyse oxygenation of ortho-hydroxychlorobiphenyls formed from PCBs by plants. Environ. Pollut. 127, 41–48 (2004). 14553993 10.1016/s0269-7491(03)00257-4

[b8] Pieper D. H. Aerobic degradation of polychlorinated biphenyls. Appl. Microbiol. Biotechnol. 67, 170–91 (2005). 15614564 10.1007/s00253-004-1810-4

[b9] Harwood C. S. & Parales R. E. The beta-ketoadipate pathway and the biology of self-identity. Annu. Rev. Microbiol. 50, 553–90 (1996). 8905091 10.1146/annurev.micro.50.1.553

[b10] Furukawa K., Tomizuka N. & Kamibayashi A. Effect of chlorine substitution on the bacterial metabolism of various polychlorinated biphenyls. Appl. Environ. Microbiol. 38, 301–310 (1979). 117752 10.1128/aem.38.2.301-310.1979 PMC243481

[b11] Bedard D. L. & Haberl M. L. Influence of chlorine substitution pattern on the degradation of polychlorinated biphenyls by eight bacterial strains. Microb. Ecol. 20, 87–102 (1990). 24193967 10.1007/BF02543870

[b12] Gilbert E. S. & Crowley D. E. Plant compounds that induce polychlorinated biphenyl biodegradation by Arthrobacter sp. Strain B1B. Appl. Environ. Microbiol. 63, 1933–1938 (1997). 9143124 10.1128/aem.63.5.1933-1938.1997 PMC168484

[b13] Tillmann S., Stroempl C., Timmis K. N. & Abraham W.-R. Stable isotope probing reveals the dominant role of Burkholderia species in aerobic degradation of PCBs. FEMS Microbiol. Ecol. 52, 207–217 (2005). 16329907 10.1016/j.femsec.2004.11.014

[b14] Kim S. & Picardal F. Microbial Growth on Dichlorobiphenyls Chlorinated on Both Rings as a Sole Carbon and Energy Source. Appl. Environ. Microbiol. 67, 1953–1955 (2001). 11282655 10.1128/AEM.67.4.1953-1955.2001 PMC92819

[b15] Ohtsubo Y., Nagata Y., Kimbara K., Takagi M. & Ohta A. Expression of the bph genes involved in biphenyl/PCB degradation in Pseudomonas sp. KKS102 induced by the biphenyl degradation intermediate, 2-hydroxy-6-oxo-6-phenylhexa-2,4-dienoic acid. Gene 256, 223–8 (2000). 11054551 10.1016/s0378-1119(00)00349-8

[b16] Uhlik O. *et al.* Plant secondary metabolite-induced shifts in bacterial community structure and degradative ability in contaminated soil. Appl. Microbiol. Biotechnol. 97, 9245–9256 (2013). 23250224 10.1007/s00253-012-4627-6

[b17] Kurzawova V. *et al.* Plant–microorganism interactions in bioremediation of polychlorinated biphenyl-contaminated soil. N. Biotechnol. 30, 15–22 (2012). 22728721 10.1016/j.nbt.2012.06.004

[b18] Leigh M. B., Fletcher J. S., Fu X. & Schmitz F. J. Root turnover: an important source of microbial substrates in rhizosphere remediation of recalcitrant contaminants. Environ. Sci. Technol. 36, 1579–83 (2002). 11999069 10.1021/es015702i

[b19] Pham T. T. M., Tu Y. & Sylvestre M. Remarkable Ability of Pandoraea pnomenusa B356 Biphenyl Dioxygenase To Metabolize Simple Flavonoids. Appl. Environ. Microbiol. 78, 3560–3570 (2012). 22427498 10.1128/AEM.00225-12 PMC3346365

[b20] Chen Y. & Murrell J. C. When metagenomics meets stable-isotope probing: progress and perspectives. Trends Microbiol. 18, 157–163 (2010). 20202846 10.1016/j.tim.2010.02.002

[b21] Neufeld J. D. *et al.* DNA stable-isotope probing. Nat. Protoc. 2, 860–866 (2007). 17446886 10.1038/nprot.2007.109

[b22] Uhlik O. *et al.* Stable isotope probing in the metagenomics era: A bridge towards improved bioremediation. Biotechnol. Adv. 31, 154–165 (2013). 23022353 10.1016/j.biotechadv.2012.09.003 PMC3578049

[b23] Leigh M. B. *et al.* Biphenyl-utilizing bacteria and their functional genes in a pine root zone contaminated with polychlorinated biphenyls (PCBs). ISME J. 1, 134–48 (2007). 18043623 10.1038/ismej.2007.26

[b24] He R. *et al.* Identification of functionally active aerobic methanotrophs in sediments from an arctic lake using stable isotope probing. Environ. Microbiol. 14, 1403–19 (2012). 22429394 10.1111/j.1462-2920.2012.02725.x

[b25] Bell T. H. *et al.* Identification of Nitrogen-Incorporating Bacteria in Petroleum-Contaminated Arctic Soils Using 15N DNA-SIP and Pyrosequencing. Appl. Environ. Microbiol. 77, 4163–4171 (2011). 21498745 10.1128/AEM.00172-11 PMC3131650

[b26] Uhlik O. *et al.* Identification of Bacteria Utilizing Biphenyl, Benzoate, and Naphthalene in Long-Term Contaminated Soil. PLoS One 7, e40653 (2012). 22808223 10.1371/journal.pone.0040653 PMC3396604

[b27] DeRito C. M., Pumphrey G. M. & Madsen E. L. Use of field-based stable isotope probing to identify adapted populations and track carbon flow through a phenol-degrading soil microbial community. Appl. Environ. Microbiol. 71, 7858–65 (2005). 16332760 10.1128/AEM.71.12.7858-7865.2005 PMC1317415

[b28] Dumont M. G. & Murrell J. C. Innovation: Stable isotope probing—linking microbial identity to function. Nat. Rev. Microbiol. 3, 499–504 (2005). 15886694 10.1038/nrmicro1162

[b29] Hernandez B. S., Koh S. C., Chial M. & Focht D. D. Terpene-utilizing isolates and their relevance to enhanced biotransformation of polychlorinated biphenyls in soil. Biodegradation 8, 153–158 (1997).

[b30] Leigh M. B. *et al.* Polychlorinated biphenyl (PCB)-degrading bacteria associated with trees in a PCB-contaminated site. Appl. Environ. Microbiol. 72, 2331–42 (2006). 16597927 10.1128/AEM.72.4.2331-2342.2006 PMC1449058

[b31] Bailey R. E., Gonsior S. J. & Rhinehart W. L. Biodegradation of the monochlorobiphenyls and biphenyl in river water. Environ. Sci. Technol. 17, 617–621 (1983). 22288707 10.1021/es00116a010

[b32] Fierer N., Bradford M. A. & Jackson R. B. Toward an ecological classification of soil bacteria. Ecology 88, 1354–1364 (2007). 17601128 10.1890/05-1839

[b33] Elliott D. R., Thomas A. D., Hoon S. R. & Sen R. Niche partitioning of bacterial communities in biological crusts and soils under grasses, shrubs and trees in the Kalahari. Biodivers. Conserv. 23, 1709–1733 (2014).

[b34] Erickson B. D. & Mondello F. J. Nucleotide sequencing and transcriptional mapping of the genes encoding biphenyl dioxygenase, a multicomponent polychlorinated-biphenyl-degrading enzyme in Pseudomonas strain LB400. J. Bacteriol. 174, 2903–2912 (1992). 1569021 10.1128/jb.174.9.2903-2912.1992 PMC205943

[b35] Glick B. R. Using soil bacteria to facilitate phytoremediation. Biotechnol. Adv. 28, 367–74 (2010). 20149857 10.1016/j.biotechadv.2010.02.001

[b36] Männistö M. K., Rawat S., Starovoytov V. & Häggblom M. M. Terriglobus saanensis sp. nov., an acidobacterium isolated from tundra soil. Int. J. Syst. Evol. Microbiol. 61, 1823–1828 (2011). 21186292 10.1099/ijs.0.026005-0

[b37] Eichorst S. A., Breznak J. A. & Schmidt T. M. Isolation and characterization of soil bacteria that define Terriglobus gen. nov., in the phylum Acidobacteria. Appl. Environ. Microbiol. doi: 10.1128/AEM.02140-06 (2007). PMC1855589 17293520

[b38] Thorenoor N., Kim Y.-H., Lee C., Yu M.-H. & Engesser K.-H. A previously uncultured, paper mill Propionibacterium is able to degrade O-aryl alkyl ethers and various aromatic hydrocarbons. Chemosphere 75, 1287–1293 (2009). 19375147 10.1016/j.chemosphere.2009.03.032

[b39] Salter S. J. *et al.* Reagent and laboratory contamination can critically impact sequence-based microbiome analyses. BMC Biol. 12, 87 (2014). 25387460 10.1186/s12915-014-0087-z PMC4228153

[b40] Singer A. C., Jury W., Luepromchai E., Yahng C. S. & Crowley D. E. Contribution of earthworms to PCB bioremediation. Soil Biol. Biochem. 33, 765–776 (2001).

[b41] Eilers K. G., Lauber C. L., Knight R. & Fierer N. Shifts in bacterial community structure associated with inputs of low molecular weight carbon compounds to soil. Soil Biol. Biochem. 42, 896–903 (2010).

[b42] Pumphrey G. M. & Madsen E. L. Field-based stable isotope probing reveals the identities of benzoic acid-metabolizing microorganisms and their *in situ* growth in agricultural soil. Appl. Environ. Microbiol. 74, 4111–8 (2008). 18469130 10.1128/AEM.00464-08 PMC2446519

[b43] Pianka E. On r-and K-selection. Am. Nat. 104, 592–597 (1970).

[b44] Margesin R., Hämmerle M. & Tscherko D. Microbial activity and community composition during bioremediation of diesel-oil-contaminated soil: Effects of hydrocarbon concentration, fertilizers, and incubation time. Microb. Ecol. 53, 259–269 (2007). 17265002 10.1007/s00248-006-9136-7

[b45] Manzoni S., Taylor P., Richter A., Porporato A. & Agren G. I. Environmental and stoichiometric controls on microbial carbon-use efficiency in soils. New Phytol. 196, 79–91 (2012). 22924405 10.1111/j.1469-8137.2012.04225.x

[b46] Margesin R. & Schinner F. Biodegradation and bioremediation of hydrocarbons in extreme environments. Appl. Microbiol. Biotechnol. 56, 650–663 (2001). 11601610 10.1007/s002530100701

[b47] Sharak Genthner B. R., Price W. A. & Pritchard P. H. Anaerobic degradation of chloroaromatic compounds in aquatic sediments under a variety of enrichment conditions. Appl. Environ. Microbiol. 55, 1466–1471 (1989). 16347940 10.1128/aem.55.6.1466-1471.1989 PMC202887

[b48] Slater H., Gouin T. & Leigh M. B. Assessing the potential for rhizoremediation of PCB contaminated soils in northern regions using native tree species. Chemosphere 84, 199–206 (2011). 21596420 10.1016/j.chemosphere.2011.04.058 PMC3502615

[b49] Wald J. *et al.* Pseudomonads Rule Degradation of Polyaromatic Hydrocarbons in Aerated Sediment. Front. Microbiol. 6, 1268 (2015). 26635740 10.3389/fmicb.2015.01268 PMC4652016

[b50] Uhlik O. *et al.* Biphenyl-Metabolizing Bacteria in the Rhizosphere of Horseradish and Bulk Soil Contaminated by Polychlorinated Biphenyls as Revealed by Stable Isotope Probing. Appl. Environ. Microbiol. 75, 6471–6477 (2009). 19700551 10.1128/AEM.00466-09 PMC2765145

[b51] He R. *et al.* Diversity of active aerobic methanotrophs along depth profiles of arctic and subarctic lake water column and sediments. ISME J. 6, 1937–48 (2012). 22592821 10.1038/ismej.2012.34 PMC3446799

[b52] He R. *et al.* Shifts in identity and activity of methanotrophs in Arctic Lake sediments in response to temperature changes. Appl. Environ. Microbiol. doi: 10.1128/AEM.00853-12 (2012). PMC3370501 22522690

[b53] Engelbrektson A. *et al.* Experimental factors affecting PCR-based estimates of microbial species richness and evenness. ISME J. 4, 642–647 (2010). 20090784 10.1038/ismej.2009.153

[b54] Schloss P. D., Gevers D. & Westcott S. L. Reducing the effects of PCR amplification and sequencing artifacts on 16S rRNA-based studies. PLoS One 6, e27310 (2011). 22194782 10.1371/journal.pone.0027310 PMC3237409

[b55] Meyer F. *et al.* The metagenomics RAST server-a public resource for the automatic phylogenetic and functional analysis of metagenomes. BMC Bioinformatics 9, 386 (2008). 18803844 10.1186/1471-2105-9-386 PMC2563014

[b56] Iwai S. *et al.* Gene-targeted-metagenomics reveals extensive diversity of aromatic dioxygenase genes in the environment. ISME J. 4, 279–285 (2010). 19776767 10.1038/ismej.2009.104 PMC2808446

[b57] Strejcek M., Wang Q., Ridl J. & Uhlik O. Hunting Down Frame Shifts: Ecological Analysis of Diverse Functional Gene Sequences. Front. Microbiol. 6, 1267 (2015). 26635739 10.3389/fmicb.2015.01267 PMC4656815

