# Supplementary material for: Synergistic Processing of Biphenyl and Benzoate: Carbon Flow Through the Bacterial Community in Polychlorinated-Biphenyl-Contaminated Soil
Source: Sci Rep. 2016 Feb 26;6:22145. doi: 10.1038/srep22145 (PMC4768254; doi:10.1038/srep22145)
Supplement: Supplementary Information [file srep22145-s1.pdf]

Synergistic Processing of Biphenyl and Benzoate: Carbon Flow Through the Bacterial Community in Polychlorinated Biphenyl Contaminated Soil

Mary-Cathrine Leewis [1]*, Ondrej Uhlik[2], Mary Beth Leigh[1]

[1] Institute of Arctic Biology, University of Alaska Fairbanks, Fairbanks, AK, USA

[2] Department of Biochemistry and Microbiology, Faculty of Food and Biochemical Technology, University of Chemistry and Technology, Prague, Czech Republic

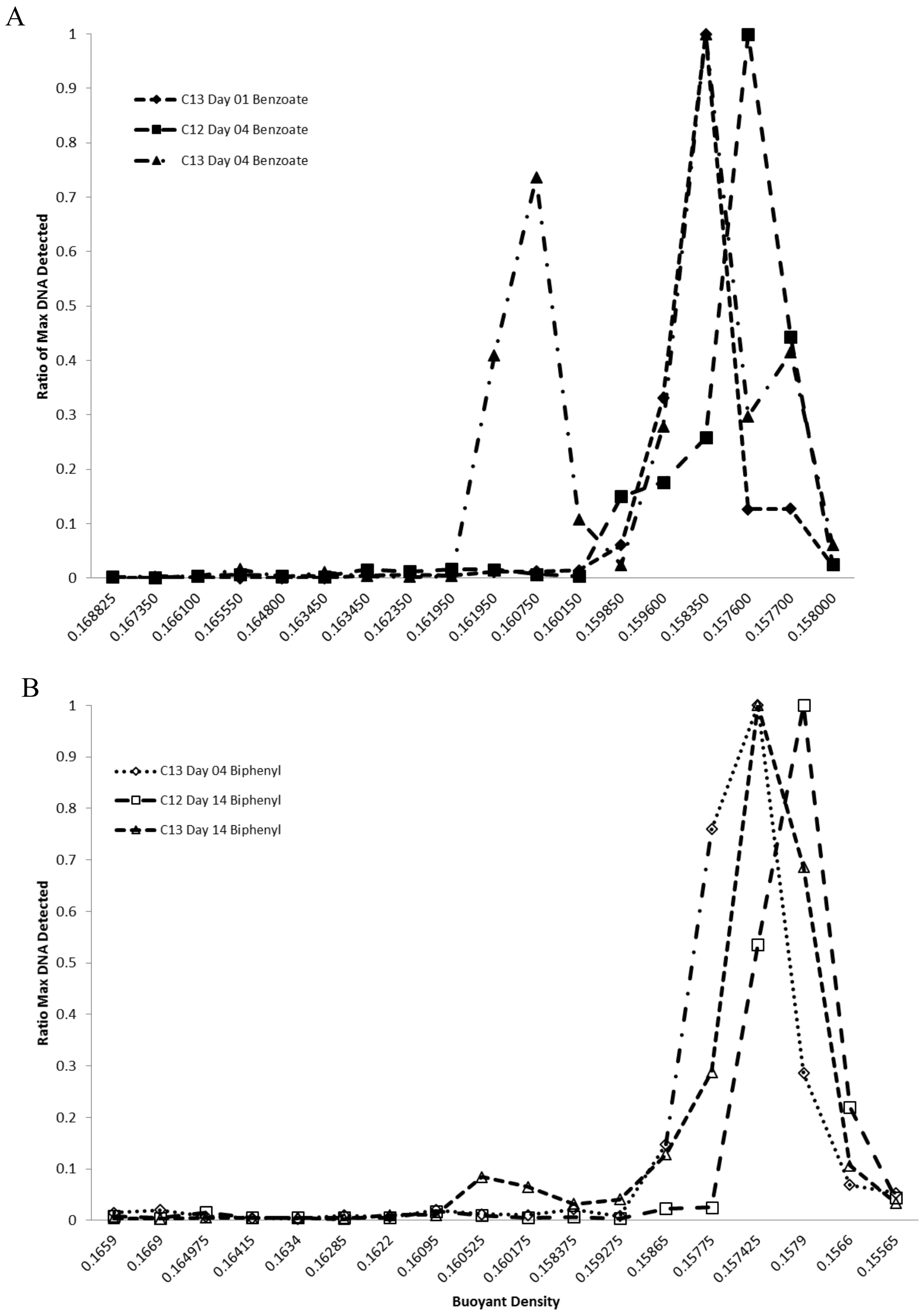

Supplementary Figure S1. Detection of ^{13}C-DNA in density gradient fractions derived from DNA in (a) ^{13}C –BZ incubated soils at 1 day, 4 days, and ^{12}C -BZ incubated control soils at day 04; (b) and ^{13}C-BP incubated soils at 4 days, 14 days, and ^{12}C -BP incubated control soils at day 14.

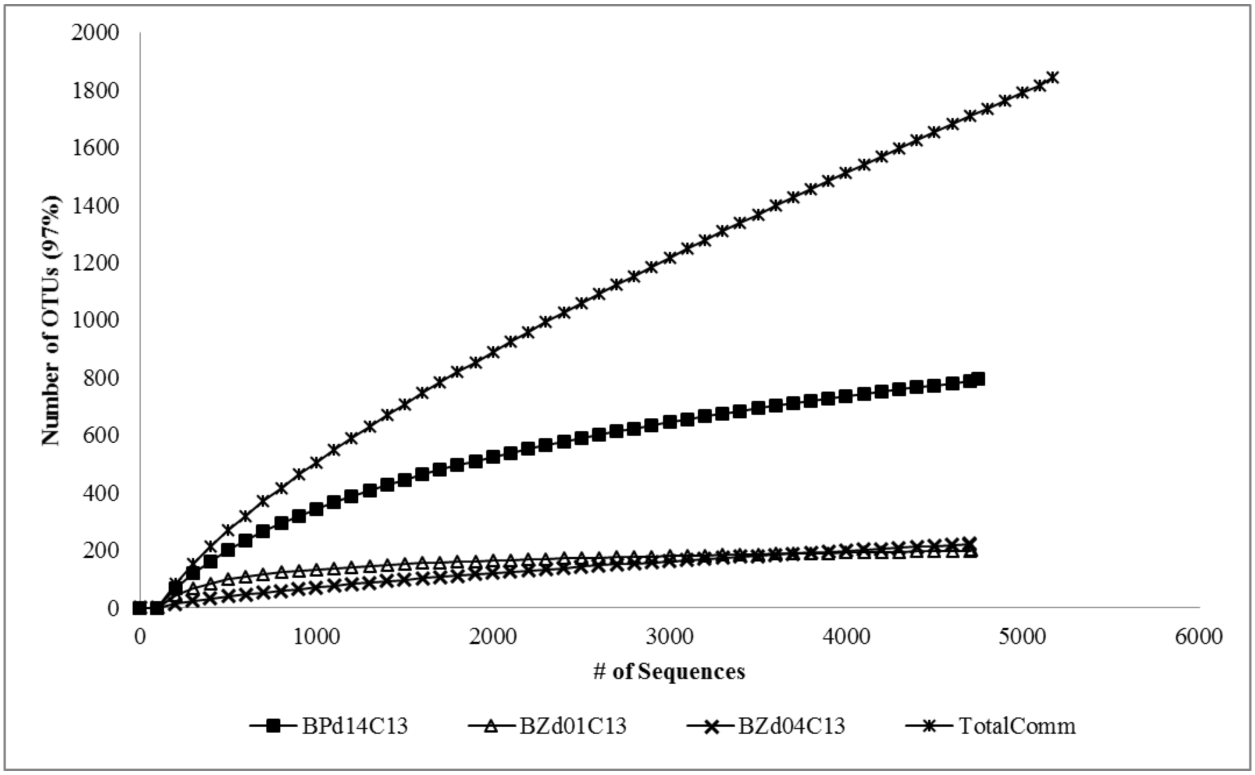

Supplementary Figure S2. Rarefaction curves for the normalized numbers of pyrosequencing reads and operational taxonomic units (OTUs) at 3% dissimilarity.

Supplementary Table S1. Significant differences among all labeled and unlabeled treatments were determined using one-way analysis of variance (ANOVA) for each incubated substrate ($p<0.05$). Presented values are p-values from the post-hoc Tukey comparison of means.

	^{13}C-Biphenyl d04	^{12}C-Biphenyl d14	^{13}C-Biphenyl d14	^{12}C-Benzoate d01	^{13}C-Benzoate d01	^{12}C-Benzoate d04	^{13}C-Benzoate d04	Time 0; Heavy Fraction	Total Community
^{13}C-Biphenyl d04		<0.01	<0.01	<0.01	<0.01	<0.01	<0.01	0.9159	0.0562
^{12}C-Biphenyl d14			<0.01	0.8672	<0.01	0.1503	<0.01	<0.01	<0.01
^{13}C-Biphenyl d14				<0.01	<0.01	<0.01	<0.01	<0.01	<0.01
^{12}C-Benzoate d01					<0.01	<0.01	<0.01	<0.01	<0.01
^{13}C-Benzoate d01						<0.01	0.9955	<0.01	<0.01
^{12}C-Benzoate d04							<0.01	<0.01	<0.01
^{13}C-Benzoate d04								<0.01	<0.01
Time 0; Heavy Fraction									0.7396
Total Community									

Supplementary Table S2. Relative abundance and phylogenetic affiliations of all pyrosequencing reads from ^{13}C-DNA obtained from SIP of PCB contaminated soil.

Phylogenetic affiliations	Biphenyl	Benzoate		Total
	Day 14	Day 01	Day 04	Community
Acidobacteria				
Acidobacteria				
Acidobacteriaceae	1.28 (69)	1.03 (134)	- (16)	2.69 (238)
unclassified	1.71 (92)	2.23 (290)	- (28)	1.87 (166)
Chloracidobacteria				
unclassified	0.48 (26)	-[b] (27)	- (19)	1.91 (169)
Solibacteres				
Solibacteraceae	3.28 (177)	0.91 (119)	- (52)	6.04 (535)
unclassified				
unclassified	1.93 (104)	2.07 (270)	- (65)	4.69 (415)
others	2.75 (148)	- (40)	- (50)	3.15 (279)
Actinobacteria				
Actinobacteria				
Corynebacteriaceae	- (0)	0.47 (61)	- (1)	- (0)
Intrasporangiaceae	1.78 (96)	0.94 (122)	- (30)	0.98 (87)
Micrococcaceae	15.28 (824)	1.26 (164)	5.48 (808)	3.56 (315)
Nocardiaceae	- (7)	11.15 (1451)	- (10)	- (27)
Nocardioidaceae	1.24 (67)	0.62 (81)	- (12)	1.72 (152)
Propionibacteriaceae	- (19)	2.46 (320)	- (30)	- (1)
Pseudonocardiaceae	0.20 (11)	0.94 (122)	- (3)	- (32)
unclassified	4.77 (257)	3.48 (472)	- (65)	7.51 (665)
others	5.06 (273)	3.96 (515)	0.52 (77)	6.50 (576)
Bacteroidetes				
Sphingobacteria				
unclassified	- (19)	- (0)	- (0)	0.50 (44)
Chloroflexi				
Ktedonobacteria				
unclassified	- (4)	- (0)	- (1)	0.67 (59)
others	4.16 (224)	2.10 (274)	- (59)	3.65 (323)
Firmicutes				
Clostridia				
Peptococcaceae	- (0)	0.51 (66)	- (1)	- (1)
Bacilli				
Staphylococcaceae	- (2)	0.58 (76)	- (2)	- (0)
Gemmatimonadetes				
Gemmatimonadetes				
Gemmatimonadaceae	4.93 (266)	1.52 (198)	- (104)	7.46 (661)
unclassified	0.91 (49)	0.25 (32)	- (7)	0.70 (62)

Nitrospirae				
Nitrospira				
Nitrospiraceae	0.52 (28)	0.23 (30)	- (9)	1.58 (140)
Proteobacteria				
Alphaproteobacteria				
Bradyrhizobiaceae	0.69 (37)	3.54 (461)	- (36)	1.95 (173)
Hyphomicrobiaceae	0.89 (48)	- (55)	- (24)	1.94 (172)
Methylobacteriaceae	- (12)	22.17 (2886)	- (0)	- (1)
Phyllobacteriaceae	- (7)	2.82 (367)	- (0)	- (18)
Sphingomonadaceae	- (13)	1.87 (244)	- (7)	- (17)
unclassified	0.54 (48)	1.81 (288)	- (18)	1.56 (138)
Betaproteobacteria				
Burkholderiaceae	0.96 (52)	- (2)	84.34 (12430)	- (5)
Comamonadaceae	0.80 (43)	- (42)	- (15)	- (32)
Oxalobacteraceae	- (11)	- (0)	- (50)	0.75 (66)
unclassified	6.29 (362)	3.00 (400)	0.74 (114)	7.80 (691)
Deltaproteobacteria				
Geobacteraceae	0.82 (44)	0.99 (129)	- (8)	0.94 (83)
Haliangiaceae	- (19)	- (18)	- (3)	- (41)
Myxococcaceae	0.48 (26)	- (0)	- (1)	- (20)
Syntrophobacteraceae	0.46 (25)	- (0)	- (23)	1.00 (89)
Gammaproteobacteria				
Enterobacteriaceae	0.76 (41)	11.85 (1543)	- (32)	- (1)
Pseudomonadaceae	6.66 (359)	1.11 (144)	- (33)	- (0)
Sinobacteraceae	2.13 (115)	0.90 (117)	- (37)	2.82 (250)
Xanthomonadaceae	4.34 (234)	0.90 (117)	0.80 (118)	0.67 (59)
unclassified				
unclassified	0.93 (50)	- (39)	- (21)	0.98 (87)
TM7				
TM7-3	5.31 (286)	- (0)	- (2)	- (1)
Others	10.00 (539)	6.01 (782)	1.10 (226)	15.37 (1361)
unclassified	4.79 (258)	4.00 (521)	0.62 (91)	6.82 (604)
Total	100 (5391)	100 (13019)	100 (14738)	100 (8856)

Taxonomic assignments were generated using the Ribosomal Database Project's classifier.

[a]The numbers of pyrosequencing reads assigned using the RDP classifier (80% confidence threshold

[b]Sequences represented < 0.5% of total sequences present in the group
